# The optimum conditions for synthesis of Fe_3_O_4_/ZnO core/shell magnetic nanoparticles for photodegradation of phenol

**DOI:** 10.1186/2052-336X-12-21

**Published:** 2014-01-09

**Authors:** Manouchehr Nikazar, Mehryana Alizadeh, Reza Lalavi, Mohammad Hossein Rostami

**Affiliations:** Department of Chemical Engineering, Amirkabir University of Technology, Tehran, Iran

**Keywords:** Fe_3_O_4_/ZnO nanoparticles, Photocatalysis, Phenol removal, Synthesis

## Abstract

The photocatalysis of phenol was studied using Fe_3_O_4_/ZnO core/shell magnetic nanoparticles (MNPs). The photocatalysts were synthesized by coating of ZnO onto the magnetite by precipitation method and characterized by XRD, SEM and FTIR measurements. Using the Taguchi method, this study analyzes the effect of parameters such as calcinations time, calcinations temperature and molar ratio of Fe_3_O_4_:ZnO on the photo activity of Fe_3_O_4_/ZnO MNPs. XRD and FTIR analysis confirm that coating process was done successfully. SEM images show that the average particle size of synthesized Fe_3_O_4_/ZnO nanoparticles was about 50 nm. The phenol removal efficiency of 88% can be achieved by using a photocatalyst which is synthesized through the optimum conditions: calcinations temperature of 550°C, calcinations time of 2 hours and molar ratio of 1:10 for Fe_3_O_4_:ZnO.

**Electronic supplementary material:**

The online version of this article (doi:10.1186/2052-336X-12-21) contains supplementary material, which is available to authorized users.

## Introduction

Semiconductor photocatalytic materials have been extensively studied in the fields of environmental purification due to their potential to destroy a wide range of pollutants at ambient temperatures and pressures, without producing harmful byproducts [1, 2]. In a photocatalytic oxidation process, organic pollutants are destroyed in the presence of semiconductor photocatalysts (e.g., TiO_2_, ZnO), an energetic light source, and an oxidizing agent such as oxygen or air [3]. Metal oxide semiconductors have been found to be the most suitable photocatalysts which is due to their photocorrosion resistance and their wide band gap energies. Among them, TiO_2_ has been studied most. On the other hand, ZnO which has high photoactivity, chemical stability, low cost and the band gap similar to TiO_2_, can be a good alternative for it [4–6]. Many types of photocatalytic reactors have been proposed according to respective application demands, but among them, a slurry type reactor is most attractive for degrading undesirable organics dissolved in water in terms of reaction surface area per unit volume of the reactor [7, 8]. However, the system using suspended photocatalyst particles requires a separation step to recover the photocatalysts. In this case, suitable techniques of high cost such as centrifugation or filtration steps are necessary to reuse fine photocatalyst particles [9]. Magnetically separable photocatalysts have attracted increasing attention due to their scientific and technological importance in the environmental purification, especially in wastewater treatment. Magnetic supports could eliminate the separation step, because the photocatalyst could be effectively recycled by applying an external magnetic field [10]. Although ZnO nanoparticles have been used as a photocatalyst [11–14], the Fe_3_O_4_/ZnO core/shell MNPs have not been sufficiently investigated [15]. In this case, both the core and the shell are of interest. The magnetic core enhancing the separation properties of suspended particles from solution and the photocatalytic properties of the outer shell zinc oxide are used to destroy organic contaminants in waste waters [16]. In this work, Fe_3_O_4_/ZnO core/shell composite catalyst was synthesized. This photocatalyst is produced by coating a layer of the zinc oxide onto the surface of magnetite core using precipitation method. The effect of parameters such as calcination temperature, calcination time, and molar ratio of Fe_3_O_4_:ZnO on the photocatalytic activity was studied by using Taguchi method. Phenol, which is well known for its biorecalcitrant and acute toxicity, was the organic matter used in this work [11, 12].

## Experimental

### Materials

Ferric chloride (FeCl_3_.6H_2_O), ferrous sulfate (FeSO_4_.7H_2_O), Zinc acetate (ZnAc_2_.2H_2_O), aqueous ammonia (NH_3_.H_2_O) and phenol (C_6_H_5_OH) were obtained from Merck company and ammonium carbonate ((NH_4_)2CO_3_) was purchased from Daejung (South Korea) and used without further purification.

### Synthesis

A co-precipitation method was used to synthesize the Fe_3_O_4_ magnetic nanoparticles (MNPs). Co-precipitation is a facile and convenient way to synthesize MNPs from aqueous salt solutions. There are three controllable parameters have been used for the synthetic procedure which each parameter has three levels (Table 1). Also, the experimental conditions are presented in Table 2[17]. This process was done by addition of ammonia to the mixture of ferric chloride (0.5 M) and ferrous sulfate (0.5 M), with molar ratio of 1.75:1 under inert argon protection, until pH value reached to 9. After 30 min stirring, the precipitate had been collected using a magnet and washed with deionized water until pH reached to 7. The modification process was accomplished via sonicating the mixture of 4 g Fe_3_O_4_ and 200 mL sodium citrate (0.5 M) for 20 min and stirring the mixture for 12 h at 60°C under argon protection. Then the precipitate was collected and rinsed with acetone. The Fe_3_O_4_/ZnO core/shell MNPs were prepared by coating the modified Fe_3_O_4_ MNPs with direct precipitation using zinc acetate and ammonium carbonate. The modified Fe_3_O_4_ was added to 100 mL of deionized water and sonicated for 20 min to make a stable ferrofluid. Then 20, 30 and 50 mL of this ferrofluid were added into a flask to form Fe_3_O_4_/ZnO composite with molar ratios of 1:6, 1:10 and 1:15 for Fe_3_O_4_:ZnO respectively. Two solutions were made by adding 12.16 g ZnAc_2_.2H_2_O and 7.6 g (NH_4_)2CO_3_ into 100 mL of deionized water respectively. These two solutions were added dropwise to the flask for each experiment. The collected precipitate was washed with water, aqueous ammonia (pH 9) and ethanol and then it was dried under vacuum for 12 h and calcined according to desired calcination temperature and time. ZnO would be produced when no ferrofluid exists in the flask.

**Table 1:** **Variable factors**

Factor	Description	Level 1	Level 2	Level 3
A	Calcination temperature (°C)	350	450	550
B	Molar ratio of Fe_3_O_4_:ZnO	1:6	1:10	1:15
C	Calcination time (h)	2	3	4

**Table 2:** **Design of experiments**

Number of experiment	Calcination temperature (°C)	Molar ratio of Fe_3_O_4_:ZnO	Calcination time (h)
1	350	1:6	2
2	350	1:10	3
3	350	1:15	4
4	450	1:6	3
5	450	1:10	4
6	450	1:15	2
7	550	1:6	4
8	550	1:10	2
9	550	1:15	3

### Characterization

In all analysis, sample number 8 was examined as Fe_3_O_4_/ZnO core/shell particles. The X-ray diffraction pattern of the samples was measured in an Equinox 3000 (Inel France). The crystallite dimensions of particles were calculated using Scherrer’s equation and nanoparticles of Fe_3_O_4_, modified Fe_3_O_4_, ZnO and Fe_3_O_4_/ZnO core/shell were examined by a Phillips scanning electron microscopy (SEM). Fourier transform infrared (FTIR) spectra obtained using a KBr method in a Perkin Elmer analyzer, USA.

### Photocatalytic tests

**Figure 1:**
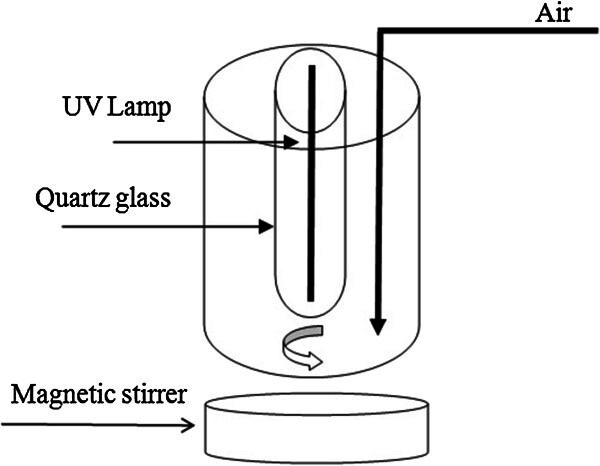
**Schematic figure of the reactor.**

Photocatalytic degradation of phenol was performed in a slurry batch reactor which consisted of cylindrical glass vessel, quartz trap, magnetic stirrer and a Philips 11 W UV-C lamp located at the center of the reactor. The schematic figure of the reactor is shown in Figure 1. In all experiments, 200 mL phenol solution (100 ppm) was taken in photocatalytic reactor and pH value of solutions had been adjusted to 5. Then 1 g/L of synthesized catalyst was added and the mixture was stirred magnetically to obtain homogeneous suspension. Before irradiation, the reaction mixture was put in darkness for 30 minutes to achieve maximum adsorption of the phenol onto the catalyst surface. After 5 h, a sample was taken and photocatalyst particles were separated using a strong magnet in a few minutes.

COD determination was utilized to analyze the samples. The COD determination tests were performed according to standard dichromate method [18]. The photodegradation efficiency was calculated from the following expression:

1 $$\eta =\left(\left(\mathrm{CO}D_{i}‒\mathrm{CO}D_{t}\right)/\mathrm{CO}D_{i}\right)*100$$

where η, COD_i_ and COD_t_ are photodegradation efficiency, initial chemical oxygen demand, and chemical oxygen demand at time t respectively.

## Results and discussion

### XRD patterns

**Figure 2:**
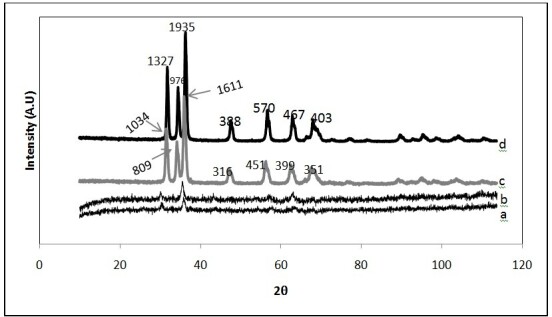
**XRD patterns of (a): modified Fe**_**3**_ **O**_**4**_ **(b): Fe**_**3**_ **O**_**4**_ **(c): ZnO (d): Fe**_**3**_ **O**_**4**_ **/ZnO core/shell.**

The X-ray diffraction patterns of samples modified Fe_3_O_4_, Fe_3_O_4_, ZnO and Fe_3_O_4_/ZnO core/shell presented in Figure 2(a-d). As it is shown in Figure 2a, the XRD peaks can match well with peaks of Fe_3_O_4_ (Figure 2b), which is in agreement with work done by Wei et al. [19]. This fact indicates that the crystalline structure of Fe_3_O_4_ MNPs can be remained after the surface modification with sodium citrate. By using Debye–Scherrer equation d = Kλ/(βcosθ), the average crystallite sized was calculated about 13.9 nm (a), 11.2 nm (b) for modified Fe_3_O_4_ and Fe_3_O_4_, respectively. Figure 2d represents the XRD pattern of Fe_3_O_4_/ZnO core/shell. Considering this figure, it is shown that after coating, we have enhancement in peak intensity which is caused by overlapping of Fe_3_O_4_ peaks. Results were obtained from Hong et al. [15] confirm these results.

### SEM images

**Figure 3:**
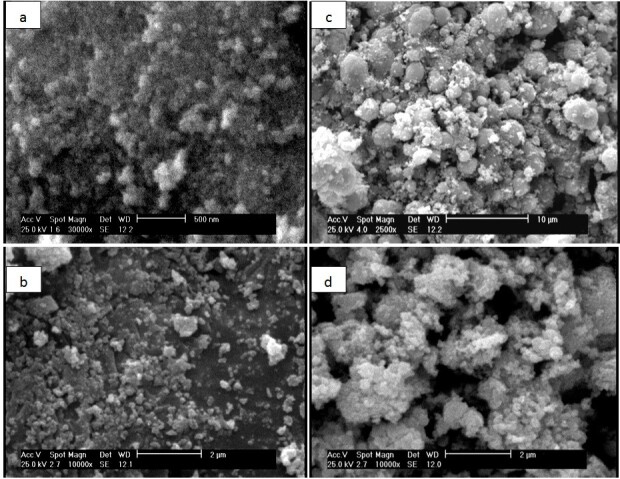
**SEM images of (a): Fe**_**3**_ **O**_**4**_ **(b): modified Fe**_**3**_ **O**_**4**_ **(c): ZnO (d): Fe**_**3**_ **O**_**4**_ **/ZnO core/shell.**

Fe_3_O_4_MNPs SEM images with sodium citrate are presented in Figure 3a and 2b before and after treatment, respectively. It is shown that the dispersion of modified iron oxide is better than unmodified one. Figure 3 represents ZnO and Fe_3_O_4_/ZnO core/shell particles. The average particle size was obtained about 57 and 48 nm, respectively.

### FTIR spectrum

**Figure 4:**
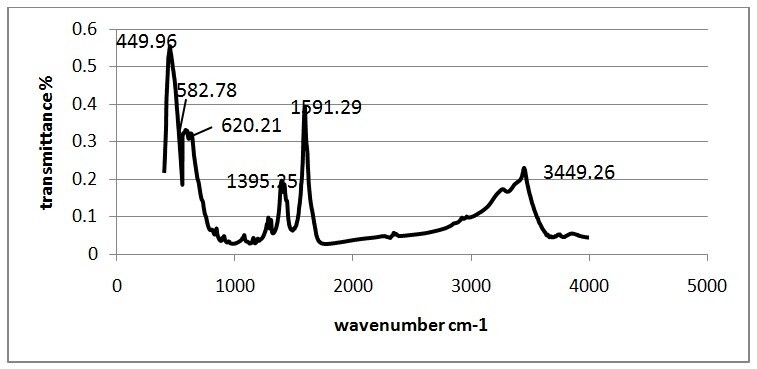
**FTIR spectrum of Fe**_**3**_ **O**_**4**_ **/ZnO nanoparticles.**

Figure 4 shows the FT-IR spectra of Fe_3_O_4_/ZnO core/shell MNPs. It can be seen that the characteristic absorption of Fe-O bond is at 582.78/cm and 620.21/cm, while that of -OH bond is at 3449.26/cm. The absorptions at 1395.25/cm and 1591.29/cm are characteristic peaks of the COO-Fe bond, which may be due to the reaction of hydroxide radical groups on the surface of Fe_3_O_4_ with carboxylate anion of sodium citrate. These peaks reveal that sodium citrate has been successfully grafted onto the surface of Fe_3_O_4_ MNPs. Also, the adsorption at 449.96 refers to Zn-O bond. Combining with XRD results, it is concluded that ZnO had been coated on the Fe_3_O_4_, successfully.

### Photoactivity

For each experiment, photo activity was examined according to photo degradation test and results are given in Table 3. It is observed that experiment number 8 has the highest phenol removal efficiency and also maximum value for mean of S/N. The aim of this study is to maximize the photodegradation of phenol, thus the higher signal to noise ratio S/N is better. Table 4 shows the mean values of S/N for all factors in their levels, $M_{\mathrm{level}}^{\mathrm{factor}}$. According to this table, optimum conditions for synthesis of catalyst are: calcination temperature of 550°C, molar ratio of 1:10 for Fe_3_O_4_: ZnO and calcination time of 2 h. At these conditions, photodegradation of 88% for 100 ppm initial phenol solution (pH = 5) with 1 mg/l catalyst is obtained after 5 hours irradiation which is comparable with results obtained by Pardeshi and Patil [11], (75.24% degradation for a 75 ppm phenol solution at pH = 5 with 1 mg/L ZnO after 8 hours sunlight irradiation) and also results obtained by Salah and his coworkers [6] (92% degradation after 5 hours irradiation of UV lamp for a 50 ppm phenol solution with 2 g/L TiO_2_). These results indicate that this novel catalyst possess high photodegradation ability and could be an effective alternative catalyst in photocatalysis of contaminated water.

**Table 3:** **The** $\left(\frac{S}{N}\right)$ **ratio of each test**

Number of experiment	Calcination temperture (°C)	Molar ratio of Fe_3_O_4_:ZnO	Time of calcination (h)	Y1%	Y2%	Y3%	$\left(\frac{S}{N}\right)$ average
1	350	1:6	2	82	83	84	38.3803
2	350	1:10	3	78	80	76	37.8362
3	350	1:15	4	57	55	56	34.9610
4	450	1:6	3	68	74	71	37.0096
5	450	1:10	4	72	73	70	37.1024
6	450	1:15	2	80	78	83	38.0895
7	550	1:6	4	62	65	65	36.1171
**8**	**550**	**1:10**	**2**	**85**	**86**	**88**	**38.7209**
9	550	1:15	3	75	76	78	37.6508

**Table 4:** $\left(\frac{S}{N}\right)$ **ratio response**

$\frac{\mathrm{factor}}{\mathrm{level}}$	${\left[{\left(\frac{S}{N}\right)}_{\mathrm{factor}}^{\mathrm{level}}\right]}_{j}$			$M_{\mathrm{factor}}^{\mathrm{level}}\;$
	**j = 1**	**j = 2**	**j = 3**	
A/1	38.3803	37.8362	34.9610	37.0592
A/2	37.0096	37.1024	38.0895	37.4005
A/3	36.1171	38.7209	37.6508	**37.4963**
B/1	38.3803	37.0096	36.1171	37.169
B/2	37.8362	37.1024	38.7209	**37.8865**
B/3	34.9610	38.0895	37.6508	36.9004
C/1	38.3803	38.0895	38.7209	**38.3969**
C/2	37.8362	37.0096	37.6508	37.4989
C/3	34.9610	37.1024	36.1171	36.0602

A: calcination temperature (°C), B: molar ratio of Fe_3_O_4_: ZnO and C: calcination time (h).

## Conclusion

Fe_3_O_4_/ZnO nanoparticles were synthesized by precipitation method. According to Taguchi method, the optimum conditions for synthesis of catalyst were achieved at 550°C for calcination temperature, 1:10 formulation of Fe_3_O_4_: ZnO, and 2 h calcinations time. XRD and FTIR analysis show that coating process was done successfully. SEM images indicate that the average particle size of synthesized Fe_3_O_4_/ZnO nanoparticles was about 48 nm. The recyclable nanoparticles exhibited good activity for the photodegradation of phenol under UV light irradiation, so that 88% removal of phenol (100 pm) is achieved after 5 h. Hence, the Fe_3_O_4_/ZnO nanopartcles could be an effective recyclable catalyst for photodegradation of phenol.

## Authors’ original submitted files for images

Below are the links to the authors’ original submitted files for images. Authors’ original file for figure 1 Authors’ original file for figure 2 Authors’ original file for figure 3 Authors’ original file for figure 4

## References

[1] Lathasree S, Nageswara RA, Sivasankar B, Sadasivam V, Rengaraj K: **Heterogeneous photocatalytic mineralization of phenols in aqueous solutions.** *J App Catal A: Environ* 2004, **223:** 101–105.

[2] Wu Y, Xing M, Zhang J, Chen F: **Effective visible light-active boron and carbon modified TiO**_**2**_**photocatalyst for degradation of organic pollutant.** *J app Catal B: Environ* 2007, **97:** 182–189.

[3] Kida T, Guan G, Yamada N, Ma T, Kimura K, Yoshida A: **Hydrogen production from sewage sludge solubilize din hot-compressed water using photocatalyst under light irradiation.** *Intern J Hydrog Eng* 2004, **29:** 269–274. 10.1016/j.ijhydene.2003.08.007

[4] Delasa H, Serrano B, Salaices M: *Photocatalytic Reaction Engineering*. United States of America: Springer Sci; 2005:1–12.

[5] Morales-Flores N, Pal U, Sanchez Mora E: **Photocatalytic behavior of ZnO and Pt-incorporated ZnO nanoparticles in phenol degradation.** *App. Cat* 2011, **394:** 269–275. 10.1016/j.apcata.2011.01.011

[6] Salah NH, Bouhelassa M, Bekkouche S, Boultif A: **Study of photocatalytic degradation of phenol.** *Desal* 2010, **166:** 338–344.

[7] Ahmed S, Rasul MG, Martens WN, Brown R, Hashib MA: **Heterogeneous photocatalytic degradation of phenols in wastewater: are view on current status and developments.** *J Desalination* 2010, **261:** 3–18. 10.1016/j.desal.2010.04.062

[8] Chung YS, Park SB, Kang DW: **Magnetically separable titania-coated nickel ferrite photocatalyst Mater.** *Chem Phys* 2005, **86:** 375–381.

[9] Kurinobu S, Tsurusaki K, Naturi Y, Kimata M, Hasegawa M: **Decomposition of pollutants in waste water using magnetic photocatalyst particles.** *J magn magn mater* 2007, **310:** 1025–1027. 10.1016/j.jmmm.2006.11.072

[10] Zhang L, Wang W, Zhou L, Shang M, Sun S: **Fe**_**3**_**O**_**4**_**coupled BiOCl: a highly efficient magnetic photocatalyst.** *App Catal B: Environ.* 2009, **90:** 458–462. 10.1016/j.apcatb.2009.04.005

[11] Pardeshi SK, Patil AB: **A simple route for photocatalytic degradation of phenol in aqueous zinc oxide suspension using solar energy.** *Sol Energy* 2008, **82:** 700–705. 10.1016/j.solener.2008.02.007

[12] Yufeng T, Mingyi Z, Yue Z, Changlu S: **TiO2 nanoparticles immobilized on polyacrylonitrile nanofibers mats: a flexible and recyclable photocatalyst for phenol degradation.** *RSC Adv* 2013, **20:** 7503–7512.

[13] Hayat K, Gondal MA, Khaled MM, Ahmed S, Shemsi AM: **Nano ZnO synthesis by modified Sol Gel method and its application in heterogeneous photocatalytic removal of phenol from water.** *App.Cat. A: General* 2011, **393:** 122–129. 10.1016/j.apcata.2010.11.032

[14] Karunkaran C, Dhanalakshmi R: **Semiconductor-catalyzed degradation of phenols with sunlight.** *Solar Energy Materials & Solar Energy Cells* 2008, **92:** 1315–1321. 10.1016/j.solmat.2008.05.002

[15] Hong RY, Zhang SZ, Di GQ, Li HZ, Zheng Y, Ding J, Wei DG: **Preparation, characterization and application of Fe**_**3**_**O**_**4**_**/ZnO core/shell magnetic nanoparticles.** *Mate Research Bull* 2008, **43:** 2457–2468. 10.1016/j.materresbull.2007.07.035

[16] Waston S, Beydoun D, Amal R: **Synthesisofa novelmagnetic photocatalyst by direct deposition of nanosized TiO2 crystal sontoa magnetic core.** *J Photochem PhotobioA: Chem* 2002, **148:** 303–313. 10.1016/S1010-6030(02)00057-6

[17] Chou Ch WC, Yeh C, Yang R, Chen J: **The optimum conditions for solid-state prepared (Y**_**3-x**_**Ce**_**x**_**) Al**_**5**_**O**_**12**_**phosphor using the taguchi method.** *Adv Pow Tech* 2012, **23:** 97–103. 10.1016/j.apt.2010.12.016

[18] Clesceri LS, Greenberg AE, Eaton AD: *Standard methods for the examination of water and wastewater*. Washington DC: 20thed Am Public Health Association; 2009.

[19] Wei Y, Han B, Hu X, Lin Y, Wang X, Deng X: **Synthesis of Fe**_**3**_**O**_**4**_**nanoparticles and their magnetic properties.** *ProcEng* 2010, **27:** 632–663.

