# Supplementary figures and images for: The optimum conditions for synthesis of Fe3O4/ZnO core/shell magnetic nanoparticles for photodegradation of phenol
Source: J Environ Health Sci Eng. 2014 Jan 9;12:21. doi: 10.1186/2052-336X-12-21 (PMC3922820; doi:10.1186/2052-336X-12-21)

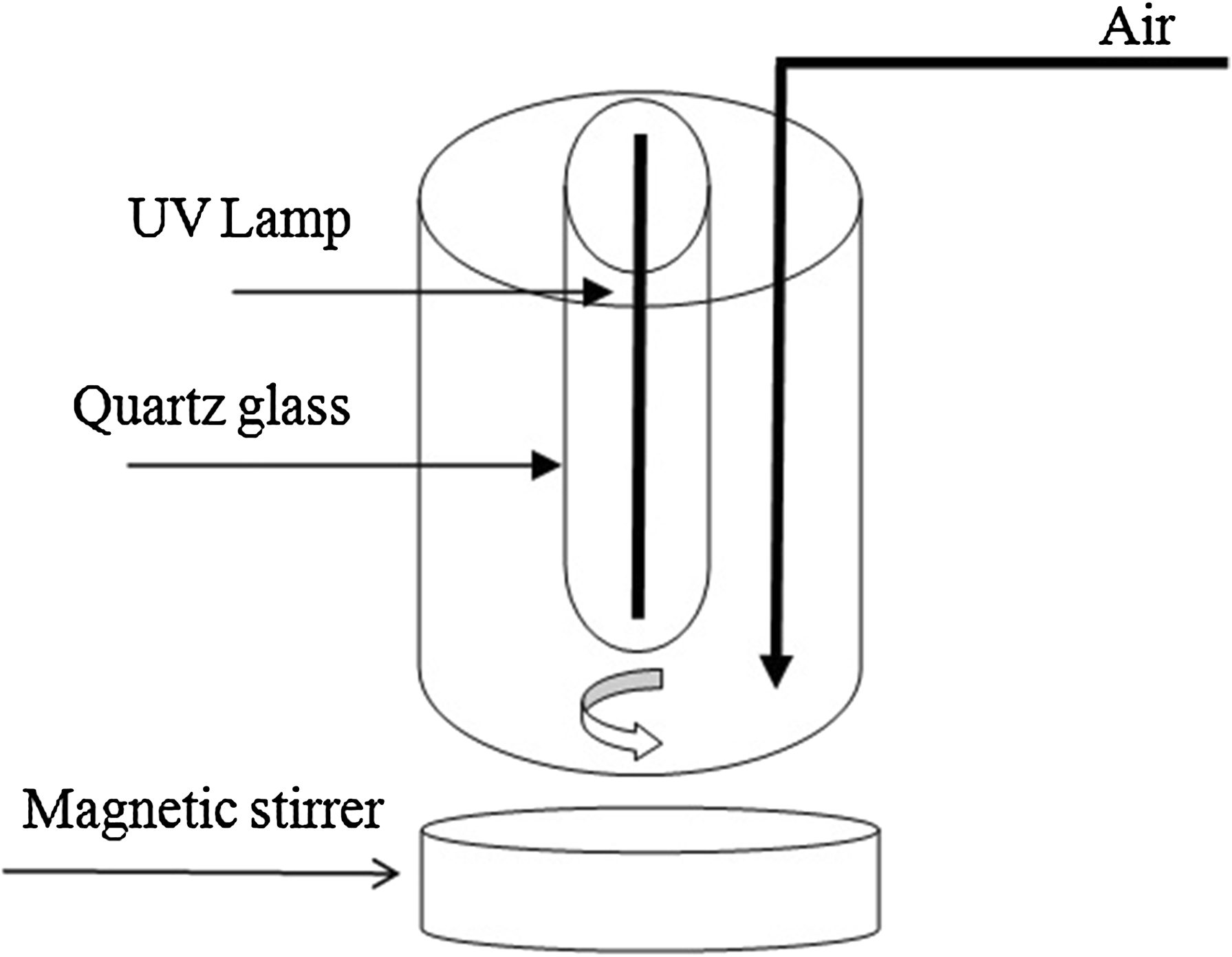

Supplement: Supplementary file 1 — Authors’ original file for figure 1 [file 40201_2013_5153_MOESM1_ESM.tif]

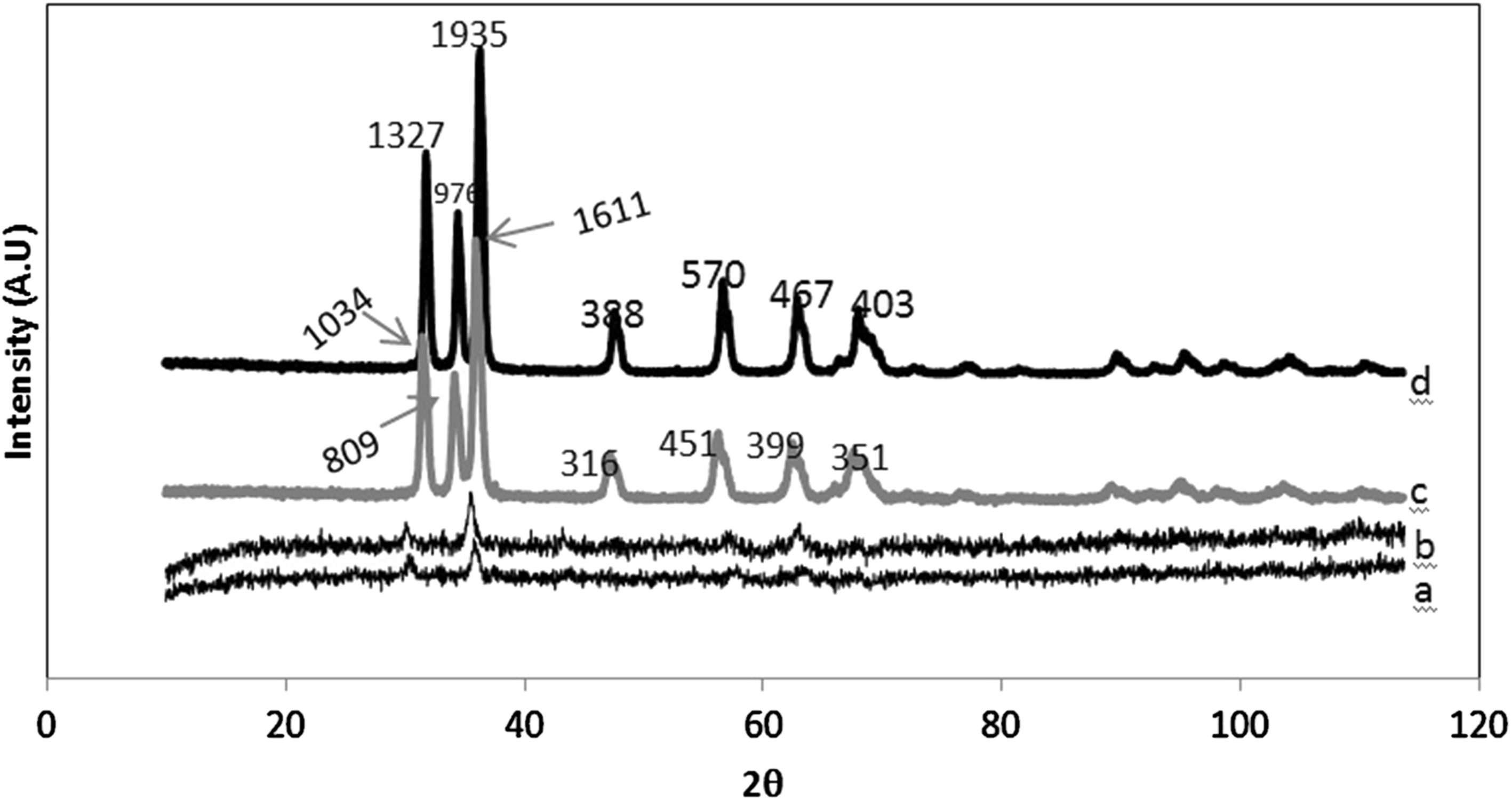

Supplement: Supplementary file 2 — Authors’ original file for figure 2 [file 40201_2013_5153_MOESM2_ESM.tif]

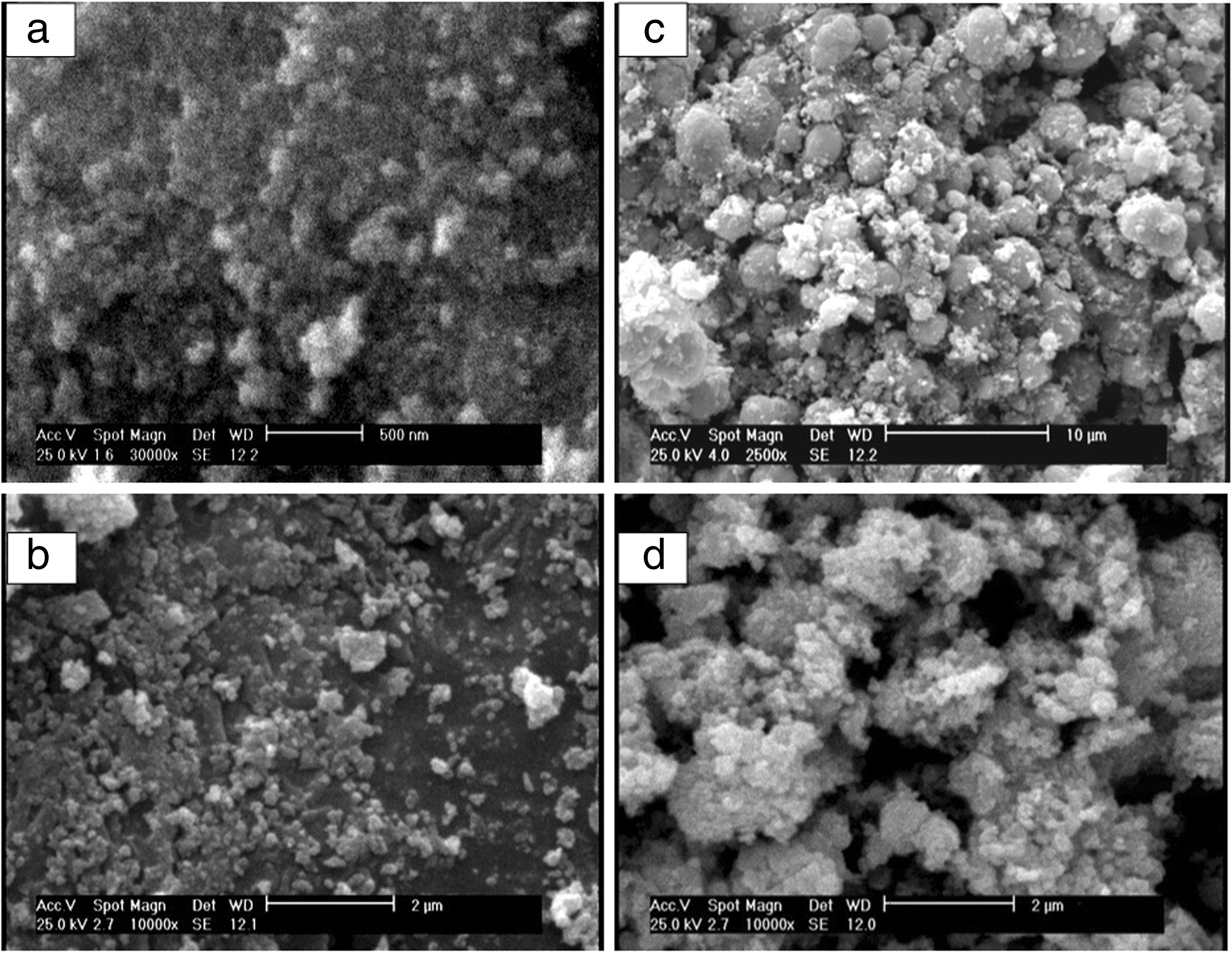

Supplement: Supplementary file 3 — Authors’ original file for figure 3 [file 40201_2013_5153_MOESM3_ESM.tif]

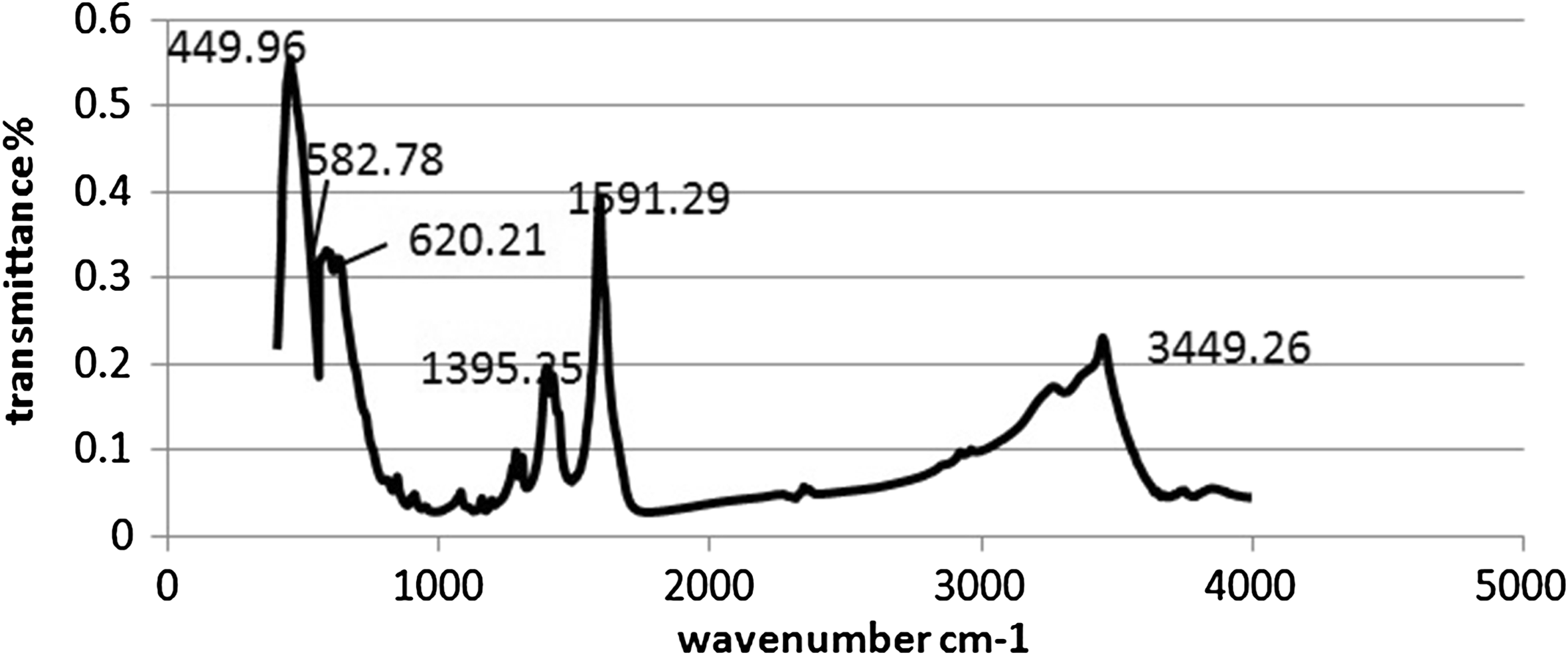

Supplement: Supplementary file 4 — Authors’ original file for figure 4 [file 40201_2013_5153_MOESM4_ESM.tif]
